# Supplementary material for: DCIS and LCIS: Are the Risk Factors for Developing In Situ Breast Cancer Different?
Source: Cancers (Basel). 2023 Sep 2;15(17):4397. doi: 10.3390/cancers15174397 (PMC10486708; doi:10.3390/cancers15174397)
Supplement: Supplementary file 1 [file cancers-15-04397-s001.zip › cancers-2505797-supplementary.pdf]

cancers

MDPI

Article

DCIS and LCIS; are the risk factors for developing in-situ breast cancer different?

Jasmine Timbres, Kelly Kohut, Michele Caneppele, Maria Troy, Marjanka K. Schmidt, Rebecca Roylance, and Elinor Sawyer

Supplementary Materials

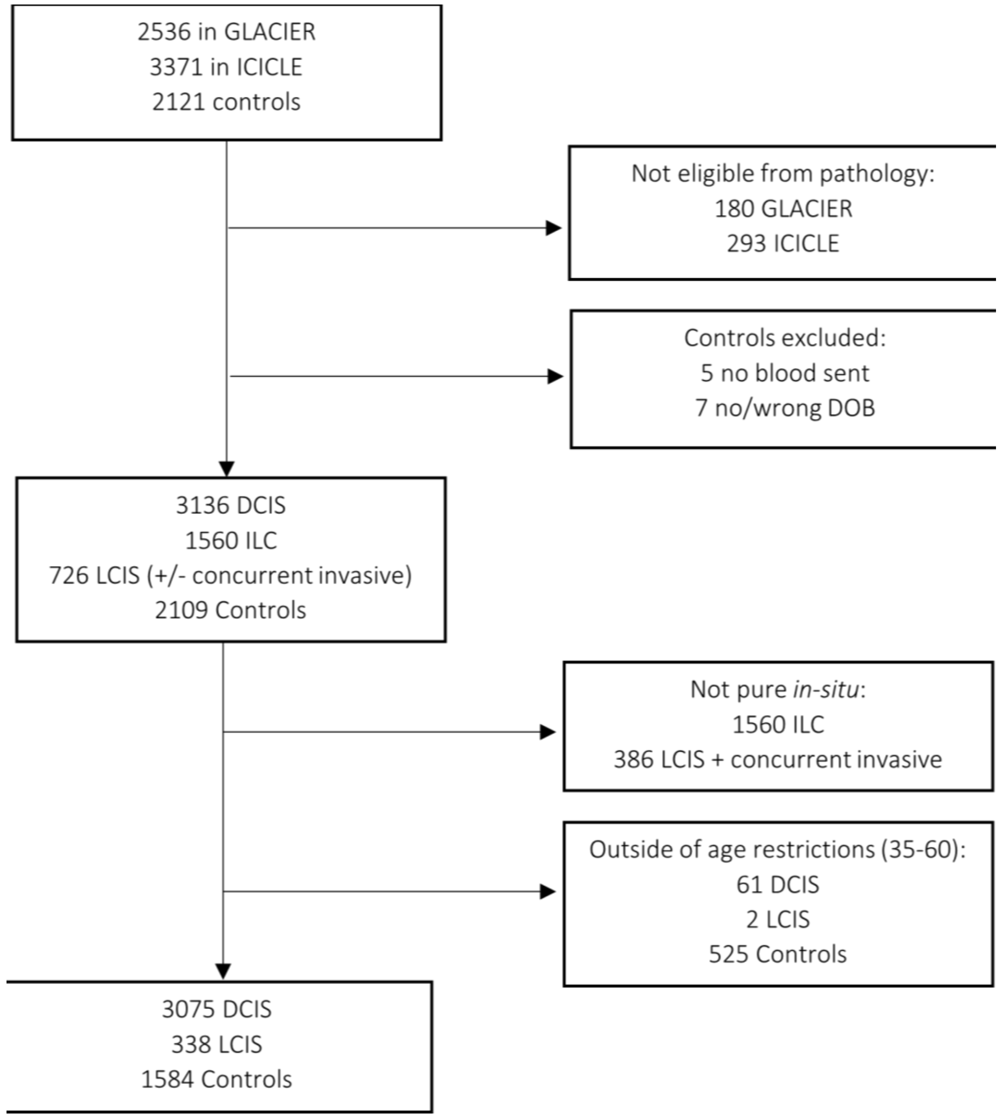

Figure S1. Flow diagram of study participant inclusions.

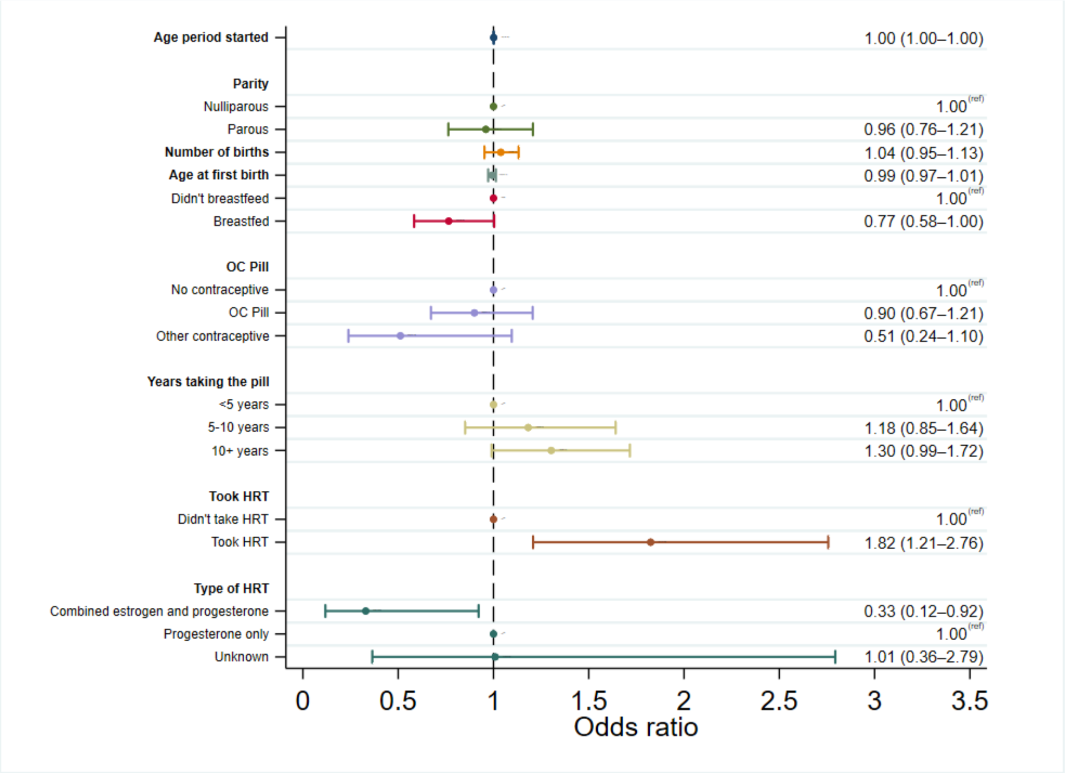

Figure S2. Logistic regression models for developing DCIS vs controls in pre-menopausal women, adjusted by age (age at diagnosis for cases, age at study entry for controls).

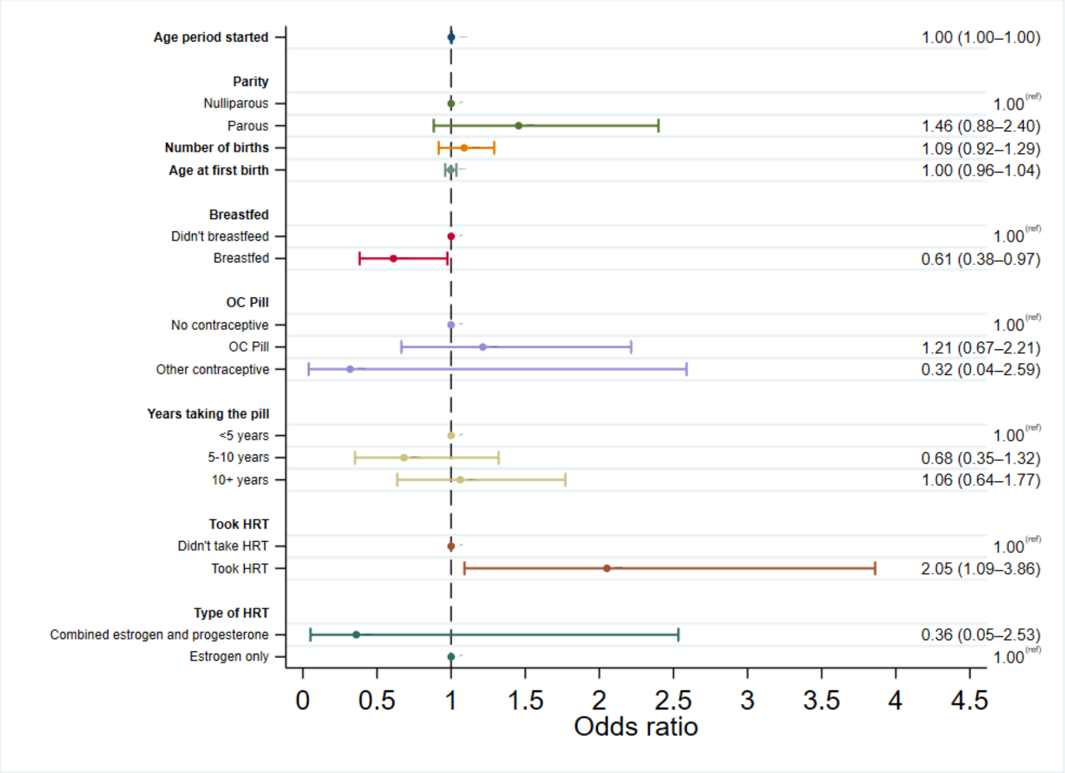

Figure S3. Logistic regression for developing LCIS vs controls in pre-menopausal women, adjusted by age (age at diagnosis for cases, age at study entry for controls).

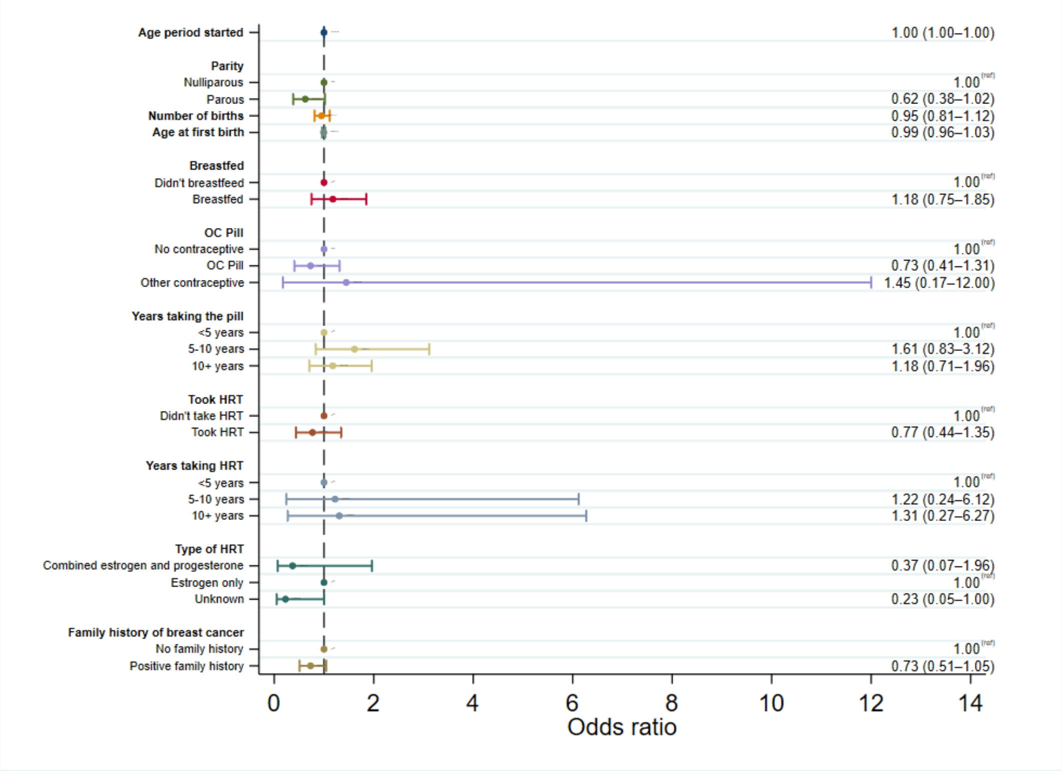

Figure S4. Logistic regression model for developing DCIS vs LCIS in pre-menopausal women, adjusted by age (age at diagnosis for cases, age at study entry for controls).

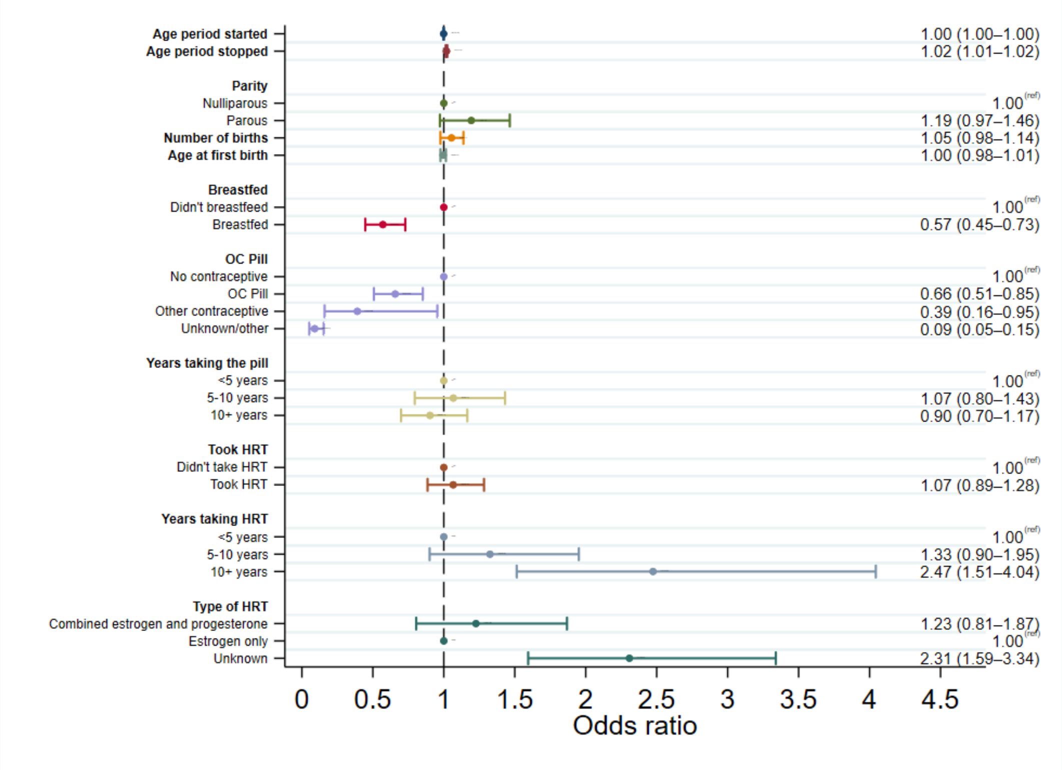

Figure S5. Logistic regression models for developing DCIS vs controls in post-menopausal women, adjusted by age (age at diagnosis for cases, age at study entry for controls).

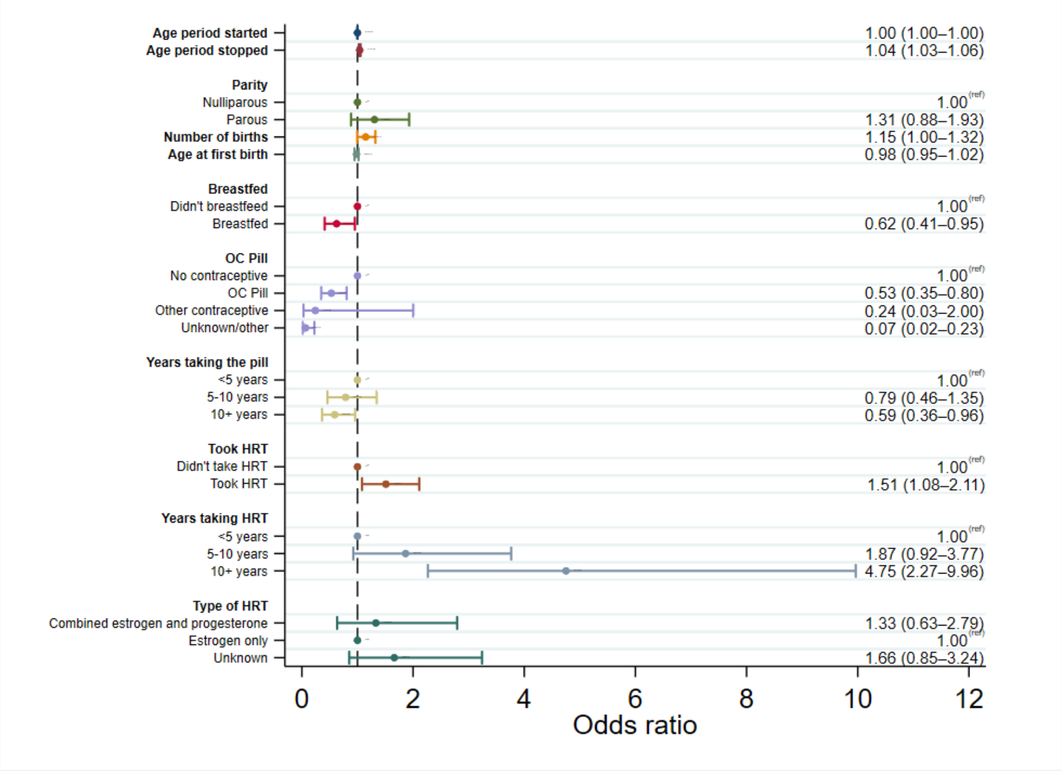

Figure S6. Logistic regression models for developing LCIS vs controls in post-menopausal women, adjusted by age (age at diagnosis for cases, age at study entry for controls).

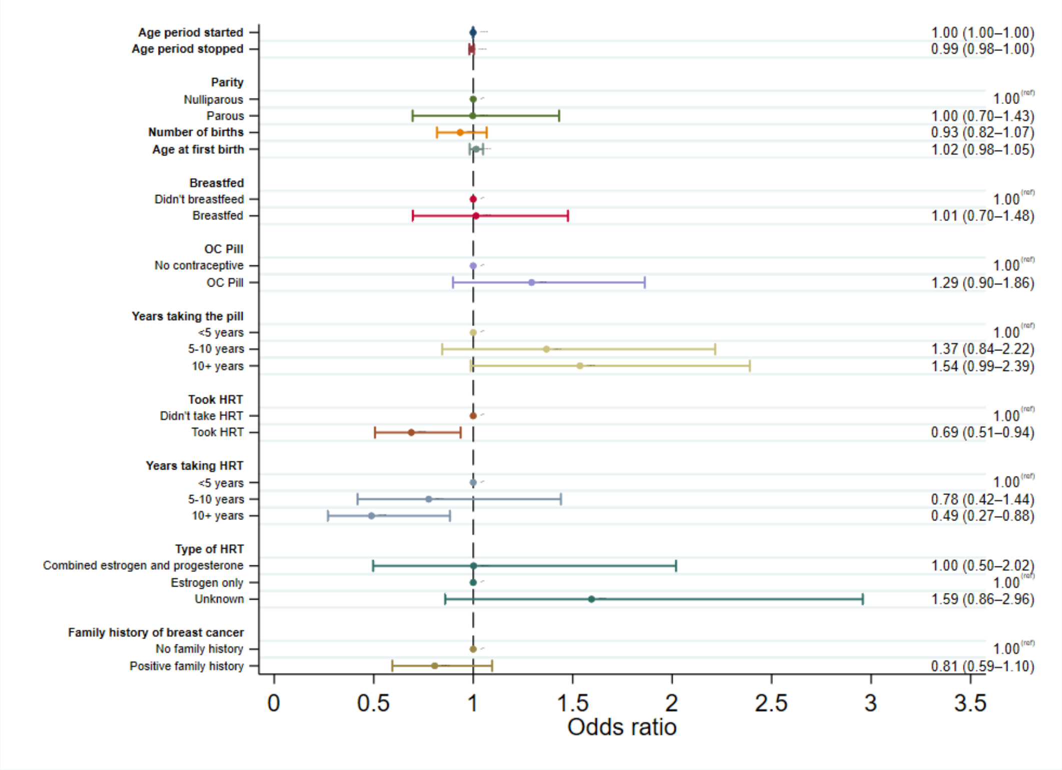

Figure S7. Logistic regression model for developing DCIS vs LCIS in post-menopausal women, adjusted by age (age at diagnosis for cases, age at study entry for controls).

Table S1. Logistic regression models for odds of developing breast cancer in all participants.

	Characteristic	OR (95%CI)		
		DCIS vs controls	LCIS vs controls	DCIS vs LCIS
	**Age period started**	**1.00 (1.00, 1.00)**	1.00 (1.00, 1.00)	**1.00 (1.00, 1.00)**
	**Parity**			
	Nulliparous	1 [ref]	1 [ref]	1 [ref]
	Parous	1.05 (0.90, 1.22)	1.29 (0.96, 1.74)	0.83 (0.62, 1.11)
In parous women	**Number of births**	1.04 (0.98, 1.1)	1.11 (1.00, 1.24)	0.94 (0.85, 1.04)
	**Age at first birth**	0.99 (0.98, 1.01)	0.99 (0.96, 1.01)	1.00 (0.98, 1.03)
	**Breastfed**			
	Didn't breastfeed	1 [ref]	1 [ref]	1 [ref]
	Breastfed	**0.65 (0.54, 0.77)**	**0.63 (0.46, 0.86)**	1.04 (0.78, 1.38)
	Unknown	1.33 (0.52, 3.40)	0.53 (0.06, 4.39)	2.61 (0.34, 20.25)
	**Contraceptive category**			
	No contraceptive	1 [ref]	1 [ref]	1 [ref]
	Took OC pill	**0.75 (0.62, 0.90)**	**0.72 (0.51, 0.99)**	1.04 (0.77, 1.41)
	Other hormonal contraceptive	**0.42 (0.24, 0.74)**	0.23 (0.05, 1.01)	1.78 (0.41, 7.73)
	Unknown	**0.22 (0.14, 0.34)**	**0.15 (0.04, 0.49)**	1.44 (0.43, 4.88)
In those who took the pill	**Years taking the pill**			
	< 5 years	1 [ref]	1 [ref]	1 [ref]
	5-10 years	1.14 (0.92, 1.41)	0.79 (0.52, 1.20)	1.41 (0.95, 2.10)
	10+ years	1.10 (0.91, 1.32)	0.84 (0.59, 1.18)	1.29 (0.93, 1.80)
	**Took HRT**			
	Didn't take HRT	1 [ref]	1 [ref]	1 [ref]
	Took HRT	**1.24 (1.05, 1.46)**	**1.62 (1.21, 2.17)**	0.77 (0.59, 1.01)
In those who took HRT	**Years taking HRT**			
	< 5 years	1 [ref]	1 [ref]	1 [ref]
	5-10 years	1.39 (0.95, 2.01)	1.84 (0.95, 3.56)	0.81 (0.45, 1.45)
	10+ years	**2.67 (1.66, 4.30)**	**4.62 (2.31, 9.28)**	**0.54 (0.31, 0.94)**
	**HRT Type**			
	Combined estrogen and progesterone	1.01 (0.69, 1.49)	1.29 (0.65, 2.54)	0.8 (0.42, 1.51)
	Estrogen only	1 [ref]	1 [ref]	1 [ref]
	Unknown/can't remember	**2.07 (1.47, 2.93)**	1.75 (0.95, 3.21)	1.22 (0.70, 2.14)
	**Age period stopped**	**1.02 (1.02, 1.02)**	**1.02 (1.02, 1.03)**	1.00 (0.99, 1.00)
	**Family history of breast cancer**			
	No family history	—	—	1 [ref]
	Positive family history	—	—	**0.77 (0.61, 0.97)**

Results where p-value <0.05 are highlighted in bold.

Table S2. Logistic regression models for odds of developing breast cancer in all pre-menopausal women.

	Characteristic	OR (95%CI)		
		DCIS vs controls	LCIS vs controls	DCIS vs LCIS
	**Age period started**	**1.00 (1.00, 1.00)**	**1.00 (1.00, 1.00)**	1.00 (1.00, 1.00)
	**Parity**			
	Nulliparous	1 ref	1 ref	1 ref
	Parous	0.96 (0.76, 1.21)	1.46 (0.88, 2.40)	0.62 (0.38, 1.02)
In parous women	**Number of births**	1.04 (0.95, 1.13)	1.09 (0.92, 1.29)	0.95 (0.81, 1.12)
	**Age at first birth**	0.99 (0.97, 1.01)	1.00 (0.96, 1.04)	0.99 (0.96, 1.03)
	**Breastfed**			
	Didn't breastfeed	1 ref	1 ref	1 ref
	Breastfed	0.77 (0.58, 1.00)	**0.61 (0.38, 0.97)**	1.18 (0.75, 1.85)
	Unknown [1]	0.89 (0.26, 3.11)	-	-
	**Contraceptive category**			
	No contraceptive	1 ref	1 ref	1 ref
	Took OC pill	0.90 (0.67, 1.21)	1.21 (0.67, 2.21)	**0.73 (0.41, 1.31)**
	Other hormonal contraceptive	0.51 (0.24, 1.10)	0.32 (0.04, 2.59)	1.45 (0.17, 12.00)
	Unknown	**5.48 (1.17, 25.7)**	—	**—**
In those who took the pill	**Years taking the pill**			
	< 5 years	1 ref	1 ref	1 ref
	5-10 years	1.18 (0.85, 1.64)	0.68 (0.35, 1.32)	1.61 (0.83, 3.12)
	10+ years	1.3 (0.99, 1.72)	1.06 (0.64, 1.77)	1.18 (0.71, 1.96)
	**Took HRT**			
	Didn't take HRT	1 ref	1 ref	1 ref
	Took HRT	**1.82 (1.21, 2.76)**	**2.05 (1.09, 3.86)**	0.77 (0.44, 1.35)
In those who took HRT	**Years taking HRT**			
	< 5 years	1 ref	1 ref	1 ref
	5-10 years	2.34 (0.57, 9.65)	1.71 (0.24, 12.34)	1.22 (0.24, 6.12)
	10+ years	7.06 (0.83, 59.8)	7.36 (0.60, 90.51)	1.31 (0.27, 6.27)
	**HRT Type**			
	Combined estrogen and progesterone	**0.33 (0.12, 0.92)**	0.36 (0.05, 2.53)	0.37 (0.07, 1.96)
	Estrogen only	1 ref	1 ref	1 ref
	Unknown/can't remember	1.01 (0.36, 2.79)	3.46 (0.68, 17.56)	0.23 (0.05, 1.00)
	**Family history of breast cancer**			
	No family history	—	—	1 ref
	Positive family history	—	—	0.73 (0.51, 1.05)

Results where p-value <0.05 are highlighted in bold.

[1] the "unknown" category of breastfeeding was omitted in the LCIS models

Table S3. Logistic regression models for odds of developing breast cancer in all post-menopausal women.

	Characteristic	OR (95%CI)		
		DCIS vs controls	LCIS vs controls	DCIS vs LCIS
	**Age period started**	1.00 (1.00, 1.00)	**1.00 (1.00, 1.00)**	1.00 (1.00, 1.00)
	**Parity**			
	Nulliparous	1 ref	1 ref	1 ref
	Parous	1.19 (0.97, 1.46)	1.31 (0.88, 1.93)	1.00 (0.70, 1.43)
In parous women	**Number of births**	1.05 (0.98, 1.14)	1.15 (1.00, 1.32)	0.93 (0.82, 1.07)
	**Age at first birth**	1.00 (0.98, 1.01)	0.98 (0.95, 1.02)	1.02 (0.98, 1.05)
	**Breastfed**			
	Didn't breastfeed	1 ref	1 ref	1 ref
	Breastfed	**0.57 (0.45, 0.73)**	**0.62 (0.41, 0.95)**	1.01 (0.70, 1.48)
	Unknown	2.62 (0.34, 20.46)	1.52 (0.09, 25.2)	1.66 (0.20, 13.55)
	**Contraceptive category**			
	No contraceptive	1 ref	1 ref	1 ref
	Took OC pill	**0.66 (0.51, 0.85)**	**0.53 (0.35, 0.80)**	1.29 (0.90, 1.86)
	Other hormonal contraceptive	**0.39 (0.16, 0.95)**	0.24 (0.03, 2.00)	1.92 (0.24, 15.17)
	Unknown	**0.09 (0.05, 0.15)**	**0.07 (0.02, 0.23)**	0.93 (0.26, 3.25)
In those who took the pill	**Years taking the pill**			
	< 5 years	1 ref	1 ref	1 ref
	5-10 years	1.07 (0.80, 1.43)	0.79 (0.46, 1.35)	1.37 (0.84, 2.22)
	10+ years	0.90 (0.70, 1.17)	**0.59 (0.36, 0.96)**	1.54 (0.99, 2.39)
	**Took HRT**			
	Didn't take HRT	1 ref	1 ref	1 ref
	Took HRT	1.07 (0.89, 1.28)	**1.51 (1.08, 2.11)**	**0.69 (0.51, 0.94)**
In those who took HRT	**Years taking HRT**			
	< 5 years	1 ref	1 ref	1 ref
	5-10 years	1.33 (0.90, 1.95)	1.87 (0.92, 3.77)	0.78 (0.42, 1.44)
	10+ years	**2.47 (1.51, 4.04)**	**4.75 (2.27, 9.96)**	**0.49 (0.27, 0.88)**
	**HRT Type**			
	Combined estrogen and progesterone	1.23 (0.81, 1.87)	1.33 (0.63, 2.79)	1.00 (0.50, 2.02)
	Estrogen only	1 ref	1 ref	1 ref
	Unknown/can't remember	**2.31 (1.59, 3.34)**	1.66 (0.85, 3.24)	1.59 (0.86, 2.96)
	**Age period stopped**	**1.02 (1.01, 1.02)**	**1.04 (1.03, 1.06)**	0.99 (0.98, 1.00)
	**Family history of breast cancer**			
	No family history	—	—	1 ref
	Positive family history	—	—	0.81 (0.59, 1.10)

Results where p-value <0.05 are highlighted in bold.

Table S4. Sensitivity analysis: logistic regression model for odds of developing *in-situ* breast cancer by HRT use excluding cases diagnosed before 2005

HRT Use	OR (95%CI)	
	DCIS	LCIS
**Took HRT**		
Didn't take HRT	1 ref	1 ref
Took HRT	1.11 (0.94, 1.32)	1.33 (0.94, 1.87)
**Years taking HRT**		
< 5 years	1 ref	1 ref
5-10 years	1.1 (0.75, 1.62)	0.99 (0.46, 2.12)
10+ years	**2.21 (1.35, 3.59)**	**2.44 (1.1, 5.42)**
Unknown	1 (0.79, 1.26)	0.82 (0.52, 1.31)

Adjusted by age at diagnosis (cases) or age at study entry (controls)
Results where p-value <0.05 are highlighted in bold.

Table S5. Logistic regression models for odds of developing *in-situ* breast cancer by contraceptive use.

Contraceptive variable	OR (95%CI)	
	DCIS	LCIS
Adjusted by age at diagnosis only (age at study entry for controls)		
**Contraceptive use**		
No contraceptive	1 ref	1 ref
Took OC pill	**0.75 (0.62, 0.90)**	**0.72 (0.51, 0.99)**
Other contraceptive	**0.42 (0.24, 0.74)**	0.23 (0.05, 1.01)
Unknown	**0.22 (0.14, 0.34)**	**0.15 (0.04, 0.49)**
**OC pill type**		
No pill	1 ref	1 ref
Estrogen & progestogen	**0.59 (0.48, 0.72)**	**0.39 (0.26, 0.59)**
Combined & progestogen only (taken separately)	**0.58 (0.4, 0.83)**	**0.23 (0.09, 0.6)**
Progestogen only	**0.49 (0.35, 0.67)**	0.68 (0.38, 1.21)
Unknown type	0.95 (0.79, 1.15)	1.08 (0.77, 1.51)
Adjusted by age and year of diagnosis (year of study entry for controls)		
**Contraceptive use**		
No contraceptive	1 ref	1 ref
Took OC pill	0.83 (0.67, 1.04)	1.11 (0.67, 1.83)
Other contraceptive	**0.38 (0.20, 0.73)**	0.54 (0.11, 2.69)
Unknown	**0.39 (0.21, 0.75)**	0.50 (0.09, 2.79)
**OC pill type**		
No pill	1 ref	1 ref
Estrogen & progestogen	0.79 (0.62, 1.01)	0.82 (0.47, 1.44)
Combined & progestogen only (taken separately)	0.79 (0.52, 1.18)	0.46 (0.16, 1.35)
Progestogen only	**0.63 (0.44, 0.92)**	1.43 (0.68, 3.02)
Unknown type	0.97 (0.77, 1.21)	1.34 (0.81, 2.21)

Results where p-value <0.05 are highlighted in bold.

Table S6. Sensitivity analysis: logistic regression model for odds of developing *in-situ* breast cancer by contraceptive use excluding cases diagnosed before 2005

Contraceptive category	OR (95%CI)	
	DCIS	LCIS
	Adjusted by age only	
No contraceptive	1 ref	1 ref
Took OC pill	**0.8 (0.66, 0.98)**	1.08 (0.71, 1.66)
Other contraceptive	**0.44 (0.24, 0.8)**	0.47 (0.11, 2.06)
Unknown	**0.19 (0.11, 0.31)**	**0.2 (0.05, 0.85)**
	Adjusted by age and year	
No contraceptive	1 ref	1 ref
Took OC pill	0.83 (0.67, 1.04)	1.11 (0.67, 1.83)
Other contraceptive	**0.38 (0.2, 0.73)**	0.55 (0.11, 2.69)
Unknown	**0.39 (0.2, 0.74)**	0.5 (0.09, 2.8)

Results where p-value <0.05 are highlighted in bold.

Table S7. Logistic regression model for odds of developing in-situ breast cancer, after matching cases and controls 1:1 on year of birth (5-year intervals) and year of study interview (exact matching)

Variable	OR (95%CI)	
	DCIS	LCIS
Adjusted by age of diagnosis for cases/entry for controls		
**Years taking HRT**		
< 5 years	1 ref	1 ref
5-10 years	1.23 (0.72, 2.10)	1.44 (0.58, 3.58)
10+ years	**2.25 (1.20, 4.21)**	**3.53 (1.23, 10.16)**
Unknown	0.86 (0.51, 1.44)	0.75 (0.28, 1.97)
**Contraceptive category**		
No contraceptive	1 ref	1 ref
Took OC pill	**0.77 (0.6, 0.99)**	0.82 (0.49, 1.36)
Other contraceptive	0.5 (0.23, 1.05)	0.47 (0.08, 2.76)
Unknown	0.76 (0.35, 1.65)	0.52 (0.12, 2.32)
Adjusted by age and year of diagnosis for cases/entry for controls		
**Contraceptive category**		
No contraceptive	1 ref	1 ref
Took OC pill	0.74 (0.55, 1.00)	0.99 (0.54, 1.82)
Other contraceptive	**0.59 (0.24, 1.45)**	1.06 (0.15, 7.70)
Unknown	**0.66 (0.25, 1.71)**	0.44 (0.07, 2.60)

Results where p-value <0.05 are highlighted in bold.

Table S8. Logistic regression models for odds of developing *in-situ* breast cancer by HRT and breastfeeding, stratified by age category (at diagnosis for cases, at study entry for controls).

Age stratification	HRT use	OR (95%CI)	
		DCIS	LCIS
<40	No	1 ref	1 ref
	Yes	**2.90 (1.11, 7.57)**	1.95 (0.22, 16.84)
40-50	No	1 ref	1 ref
	Yes	**1.57 (1.09, 2.24)**	**2.45 (1.45, 4.13)**
50-60	No	1 ref	1 ref
	Yes	1.18 (0.98, 1.44)	**1.59 (1.11, 2.29)**

Age stratification	Breastfed	OR (95%CI)	
		DCIS	LCIS
<40	No	1 ref	1 ref
	Yes	1.55 (0.79, 3.03)	1.84 (0.40, 8.56)
40-50	No	1 ref	1 ref
	Yes	**0.71 (0.53, 0.95)**	**0.56 (0.35, 0.90)**
50-60	No	1 ref	1 ref
	Yes	**0.48 (0.37, 0.62)**	**0.49 (0.31, 0.79)**

Results where p-value <0.05 are highlighted in bold.

Table S9. Sensitivity analysis: logistic regression model for odds of developing *in-situ* breast cancer by type of oral contraceptive, excluding cases diagnosed before 2005

Type of pill	OR (95%CI)	
	DCIS	LCIS
No pill	1 ref	1 ref
Estrogen & progestogen	0.79 (0.63, 1.01)	0.82 (0.46, 1.43)
Combined & progestogen only (taken separately)	0.78 (0.52, 1.17)	0.46 (0.16, 1.36)
Progestogen only	**0.63 (0.44, 0.92)**	1.43 (0.68, 3.01)
Unknown type	0.97 (0.77, 1.21)	1.34 (0.81, 2.21)

Adjusted by age at diagnosis (cases)/ age at study entry (controls), and year at diagnosis (cases)/year at study entry (controls)
Results where p-value <0.05 are highlighted in bold.

Table S10. Multinomial logistic regression models for odds of developing DCIS histopathological subtype (by nuclear grade or estrogen receptor status)

Histopathological subtype		OR (95%CI)
	By nuclear grade	
**Low grade**	No contraceptive	1 ref
	Took OC pill	0.76 (0.53-1.09)
	Other contraceptive	0.14 (0.02-1.1)
	Unknown	0.35 (0.1-1.28)
**Intermediate grade**	No contraceptive	1 ref
	Took OC pill	0.83 (0.63-1.1)
	Other contraceptive	**0.29 (0.1-0.82)**
	Unknown	**0.3 (0.11-0.79)**
**High grade**	No contraceptive	1 ref
	Took OC pill	0.85 (0.67-1.07)
	Other contraceptive	**0.43 (0.21-0.88)**
	Unknown	**0.36 (0.17-0.74)**
**Grade missing**	No contraceptive	1 ref
	Took OC pill	0.83 (0.56-1.21)
	Other contraceptive	0.62 (0.19-2.04)
	Unknown	1.26 (0.49-3.24)
	By estrogen receptor status	
**ER-**	No contraceptive	1 ref
	Took OC pill	**0.68 (0.49-0.96)**
	Other contraceptive	0.37 (0.1-1.31)
	Unknown	**0.19 (0.04-0.85)**
**ER+**	No contraceptive	1 ref
	Took OC pill	0.85 (0.66-1.08)
	Other contraceptive	**0.19 (0.07-0.52)**
	Unknown	**0.31 (0.14-0.7)**
**ER Missing**	No contraceptive	1 ref
	Took OC pill	0.86 (0.68-1.1)
	Other contraceptive	0.56 (0.27-1.16)
	Unknown	0.54 (0.26-1.12)

Adjusted by age at diagnosis (cases)/ age at study entry (controls), and year at diagnosis (cases)/year at study entry (controls)
Results where p-value <0.05 are highlighted in bold.

Appendices

Study Questionnaires:

Appendix S1: ICICLE study questionnaire
Appendix S2: GLACIER study
questionnaire Appendix S3: Control
questionnaire

Appendix S1: ICICLE study questionnaire

ICICLE – A study to Investigate the genetiCs of In situ Carcinoma of the ductaL subtypE,
Page 1 of 3 (version 1.1 8th March 2008)

Your name ....…………………….............. **Date of birth** .... ……………………..

Ethnic Origin

Please tick the box of the ethnic group you feel you belong to:

White
British ☐
Irish ☐
Other White ☐ Details:…………………………………………………

Mixed
White/Black Caribbean ☐
White/Black African ☐
White/Asian ☐
Other Mixed ☐ Details…………………………………………………

Asian or Asian British
Indian ☐
Pakistani ☐
Bangladeshi ☐
Other Asian ☐ Details…………………………………………………

Black or Black British
Caribbean ☐
African ☐
Other Black ☐

Chinese ☐

Other ☐ Details…………………………………………………

Height……………… **Weight………………**

Do you smoke? yes ☐ **no** ☐

If yes – how much do you smoke?………………………………………………..

and for how long have you smoked?……………………………………………..

What is your average alcohol intake per week?…………………………………

Please provide details of *any* breast lumps or breast operations and in particular whether surgery removed a part or the whole breast (Please write what you can remember about the date, hospital and doctor.)

....

....

....

....

Following surgery did you require any additional treatment e.g. radiotherapy and/or tablets?

....

If you did receive tablets was this as part of a clinical trial?

....

Have you had any other benign tumours (non-cancer) or cancers?

....

....

Have you ever had genetic counselling? If so, where, when and with whom?

....

....

Has any blood relative in your family had a cancer or a benign tumour of the breast removed or had cancer elsewhere in the body? If so, please list these people (as best you can), but <u>without</u> giving their names (for example: sister, breast cancer at age 65, or mother, fibroids at age 45). Please write exactly how the person is related to you.

....

....

....

How old were you when your periods started? …………………………………..

Are you still having periods? yes ☐ no ☐

- ***IF NO**, at what age did your periods stop?*..

Did they stop naturally?…………………………………………………………..
If they did not stop naturally, how did they stop?………………………………………………

- ***IF YES**, are your cycles still regular?*…………………………………………….

Did you ever use birth control pills? yes ☐ no ☐

- **IF YES,** what was the type and for how long?………………………………………………………………

Have you ever had fertility treatment (to help get pregnant)? yes ☐ no ☐

- **IF YES,** what was the treatment and for how long?

………………………………………………………………

Have you ever been pregnant? yes ☐ no ☐

- **IF YES,** how old were you at each pregnancy and what happened at the end of the pregnancy (for example, baby was born, miscarriage, abortion)?

……………………………………………………………………..

……………………………………………………………………..…………...

……………………………………………………………………..…………...

If you have had children did you breastfeed? yes ☐ no ☐

- **IF YES**, how many children and for how long?………………………………

……………………………………………………………………..…………...

……………………………………………………………………..…………...

Did you ever take hormone replacement therapy? yes ☐ no ☐
(hormone pills after your periods stopped)

- **IF YES,** what type and for how long?

……………………………………………………………………..…………...

Appendix S2: GLACIER study questionnaire

GLACIER – A study to investigate the Genetics of Lobular Carcinoma *In situ* in Europe, Page 1 of 3 (version 3, 21 March 2007)

Your name ....… **Date of birth** .... ……………………...

Ethnic Origin

Please tick the box of the ethnic group you feel you belong to:

White
British ☐
Irish ☐
Other White ☐ Details:………………………………………………

Mixed
White/Black Caribbean ☐
White/Black African ☐
White/Asian ☐
Other Mixed ☐ Details………………………………………………….

Asian or Asian British
Indian ☐
Pakistani ☐
Bangladeshi ☐
Other Asian ☐ Details………………………………………………….

Black or Black British
Caribbean ☐
African ☐
Other Black ☐

Chinese ☐

Other ☐ Details………………………………………………

Have you had *any* breast lumps or breast operations? (If a lump was removed, please write what you can remember about the date, hospital and doctor)

....……

....……

....……

....……

....……

Approved by Southampton & South West Hampshire Research Ethics Committee No 06/Q1702/64

Have you had any other benign tumours (non-cancer) or cancers?

....

....

....

Have you ever had genetic counselling? If so, where, when and with whom?

....

Has any blood relative in your family had a cancer or a benign tumour of the breast removed or had cancer elsewhere in the body? If so, please list these people (as best you can), but <u>without</u> giving their names (for example: sister, breast cancer at age 65, or mother, fibroids at age 45).

Please write exactly how the person is related to you.

....

....

....

....

How old were you when your periods started? …………………………………..

Are you still having periods? yes ☐ no ☐

- ***IF NO****, at what age did your periods stop?..*
  Did they stop naturally?…………………………………………………………...
  How did they stop?………………………………………………………………….

- ***IF YES****, are your cycles still regular?…………………………………………….*

Did you ever use birth control pills? yes ☐ no ☐

- **IF YES,** what was the type and for how long?…………………………………………

Have you ever had fertility treatment (to help get pregnant)? yes ☐ no ☐

- **IF YES,** what was the treatment and for how long?…………………………………..

Have you ever been pregnant? yes ☐ no ☐

- **IF YES,** how old were you at each pregnancy and what happened at the end of the pregnancy (for example, baby was born, miscarriage, abortion)? ………………………………………………………………………………..

…………………………………………………………………………………..…………....

…………………………………………………………………………………..…………....

If you have had children did you breastfeed? yes ☐ no ☐

- **IF YES**, how many children and for how long?……………………………….

…………………………………………………………………………………..…………....

…………………………………………………………………………………..…………....

Did you ever take hormone replacement therapy? yes ☐ no ☐
(hormone pills after your periods stopped)

- **IF YES,** what type and for how long?

…………………………………………………………………………………..…………....

Appendix S3: Control questionnaire

SHORT QUESTIONNAIRE FOR CONTROLS AND UNAFFECTED RELATIVES

Page 1 of 2 (version 3, 21st March 2007)

Your name ....…………………….............. **Date of birth** .... ……………………...

Ethnic Origin...................…………………….............

Please tick the box of the ethnic group you feel you belong to:

White
British ☐
Irish ☐
Other White ☐ Details:………………………………………………

Mixed
White/Black Caribbean ☐
White/Black African ☐
White/Asian ☐
Other Mixed ☐ Details……………………………………………….

Asian or Asian British
Indian ☐
Pakistani ☐
Bangladeshi ☐
Other Asian ☐ Details……………………………………………….

Black or Black British
Caribbean ☐
African ☐
Other Black ☐

Chinese ☐

Other ☐ Details………………………………………………

I confirm that none of the following relatives have had lobular carcinoma *in situ* (LCIS) of the breast or breast cancer (Please initial each box)

Parents ☐

Brothers or sisters (if any) ☐

Children (if any) ☐

Uncles or aunts (if any) ☐

Please answer the following questions:

How old were you when your periods started? ………………………………..

Are you still having periods? yes ☐ no ☐

- ***IF NO**, at what age did your periods stop?*..
  Did they stop naturally?………………………………………………………...
  How did they stop?…………………………………………………………………
- ***IF YES**, are your cycles still regular?*…………………………………………….

Did you ever use birth control pills? yes ☐ no ☐

- **IF YES,** what was the type and for how long?…………………………………………

Have you ever had fertility treatment (to help get pregnant)? yes ☐ no ☐

- **IF YES,** what was the treatment and for how long?………………………………..

Have you ever been pregnant? yes ☐ no ☐

- **IF YES,** how old were you at each pregnancy and what happened at the end of the pregnancy (for example, baby was born, miscarriage, abortion)?……………………………………………………………………….

……………………………………………………………………..……………....

……………………………………………………………………..……………....

If you have had children did you breastfeed? yes ☐ no ☐

- **IF YES**, how many children and for how long?……………………………….

……………………………………………………………………..……………....

……………………………………………………………………..……………....

Did you ever take hormone replacement therapy? yes ☐ no ☐
(hormone pills after your periods stopped)

- **IF YES,** what type and for how long?

……………………………………………………………………..……………....
